# Additive Manufacturing of a PA11 Prototype Fabricated via Selective Laser Sintering for Advanced Industrial Applications

**DOI:** 10.3390/polym17233111

**Published:** 2025-11-24

**Authors:** Giovanna Colucci, Domenico Riccardi, Alberto Giubilini, Massimo Messori

**Affiliations:** 1 Department of Applied Science and Technology (DISAT), Politecnico di Torino, Corso Duca degli Abruzzi 24, 10129 Torino, Italy; massimo.messori@polito.it; 2 ZECA S.p.A., Strada della Chiara 25, 10080 Feletto Canavese, Torino, Italy; domenico.riccardi@zeca.it; 3 Department of Management and Production Engineering (DIGEP), Politecnico di Torino, Corso Duca degli Abruzzi 24, 10129 Torino, Italy; alberto.giubilini@polito.it

**Keywords:** polyamide 11 (PA11), Additive Manufacturing (AM), selective laser sintering (SLS), prototype

## Abstract

Selective Laser Sintering (SLS) is an Additive Manufacturing (AM) technology that is receiving considerable attention in the scientific and industrial communities due to its great ability to efficiently produce functional and complex parts. The present work aims to fabricate a real prototype via SLS, such as a hose reel for industrial applications, using polyamide 11 (PA11) as a starting material. Characterization of the PA11 powder properties was first carried out from a thermal and morphological viewpoint to determine the powder’s thermal stability by TGA, the sintering window and degree of crystallinity by DSC, and the microstructure by SEM, PSD, and XRD analyses. The results revealed that PA11 has a 45-micron average particle size, circularity close to 1, and a Hausner ratio of 1.17. Together, these parameters ensure that PA11 powder flows smoothly, packs uniformly, and forms dense and defect-free layers during the SLS process, directly contributing to high part quality, dimensional precision, and stable process performance. The printability of the PA11 was optimized for the realization of 3D-printed parts for industrial applications. Finally, the quality of the printed samples and the mechanical and thermal performance were investigated. Several PA11-based parts were fabricated via SLS, showing a high level of complexity and definition, ideal for industrial applications, as confirmed by the predominantly green areas of the colored maps of X-CT. A complete prototypal case for a hose reel was assembled by using the parts realized, and it was chosen as a technological demonstrator to verify the feasibility of PA11 powder in the production of industrial professional components.

## 1. Introduction

Selective Laser Sintering (SLS) is an Additive Manufacturing (AM) technology that has found extensive applications within numerous industrial sectors. SLS involves the use of a laser to selectively fuse powdered material to realize, layer by layer, a 3D-printed component based on a previously selected 3D model. The process allows the production of parts with complex geometries that would be difficult or impossible to obtain with traditional manufacturing methods [1,2]. Specifically in industrial applications, SLS has proven to be particularly valuable for rapid prototyping, small-batch production, and the realization of customized parts with specific detailed architectures [2].

Moreover, its ability to reduce raw material consumption, reduce waste, lower tooling costs, and offer high design flexibility has made it a key tool in industries such as aerospace, automotive, healthcare, and consumer goods. By leveraging the precision and high versatility of SLS, manufacturers can enhance product performance, shorten time-to-market, and lower production costs, making SLS a pivotal technology for modern industrial manufacturing [3,4].

Together with the above-mentioned advantages, SLS technology also guarantees the production of components with high dimensional accuracy and surface quality, without the need for costly molds and support structures. This is possible because the un-sintered powder surrounding the 3D-printed part acts as a natural support, often eliminating the need for further post-processing after part removal [5,6,7].

However, there is a limited list of polymers commercially available in the form of powder for SLS applications, which mainly includes thermoplastic polymers such as polyamide 11 (PA11) and polyamide 12 (PA12), acrylonitrile butadiene styrene (ABS), and polypropylene (PP) widely used for prototyping applications and the fabrication of custom parts for many industrial purposes [8,9,10]. At the same time, there is an urgent need for designing, developing, and manufacturing more sustainable materials with lower environmental impact.

For this reason, the development and use of bio-based and/or biodegradable polymer materials as alternatives to traditional fossil-based plastics is of crucial importance for industrial applications [4,5,6,7]. In this scenario, the use of PA11 in SLS can represent a significant advancement in both sustainable manufacturing and AM technologies.

PA11 derives from renewable sources, like castor oil, an important feedstock highly demanded in industry due to its renewability, that offers a valid alternative to the polymers traditionally used in SLS processes.

A further characteristic of PA11 compared to other polyamides is its low moisture absorption, yielding good dimensional stability. Moreover, its exceptional mechanical properties, including high strength, durability, flexibility, and resistance to chemicals, make bio-based PA11 an ideal candidate for producing high-performance parts through AM [11,12].

In this context, PA11 powder is selectively sintered by a laser to create complex geometries and functional components, with applications ranging from automotive parts to medical devices and consumer products. The material’s bio-based nature contributes to its reduced environmental impact, aligning with the growing demand for sustainable manufacturing solutions. PA11 not only offers improved mechanical properties but also a more environmentally friendly lifecycle, starting from its production up to its disposal [13,14,15].

Recent studies are reported in the literature that address advancements in PA11 for SLS applications, including works on PA11 powder recyclability, process optimization, and mechanical performance investigation [13,16,17,18].

Some authors have studied the effect of reusing PA11 powder in the SLS process, evaluating the number of times it can be reused by considering the dimensional accuracy and the thermal and tensile properties of the recycled powder. They found that from 100% virgin powder to the third reuse of the PA11 powder, there is a decrease in powder wastage, crystallinity, and tensile strength. However, from the fourth use, the tensile properties of the sintered PA11 sample significantly degrade, negatively affecting the brittleness and ductility [13]. Another study focused on extending the accuracy optimization to other design features to enhance the appearance of the parts by considering the position of the part in the powder bed, the build setup, and the presence of thin walls in the final components, employing a Taguchi method to study their correlations [16].

Conversely, Yao et al. [17] observed that when PA11 powder was reused in SLS, the sintered parts showed lower density due to increased void content and changes in crystallinity in the sintered part, which then compromises their final mechanical performance. Overall, the focus of these investigations has been on producing small, printed specimens, with limited attention given to larger or industrial-scale parts.

In this scenario, the novelty of this research lies in the fabrication, via SLS, of a fully 3D-printed functional prototype of an industrial hose reel by using PA11 as an excellent choice for industries aiming to balance performance with sustainability. Furthermore, the use of PA11 in SLS enables industries to manufacture lightweight, durable, and custom parts while reducing their reliance on fossil fuels, supporting the circular economy as PA11 is biodegradable and can be recycled more easily than many other materials. Several PA11-based components were successfully fabricated with a high level of printing definition and dimensional accuracy, suitable to be assembled in a prototype hose reel for a real industrial application.

## 2. Materials and Methods

### 2.1. Materials

Aurora PA11-based material is a commercially available bio-based PA11 thermoplastic powder purchased from Wematter 3D (Wematter, Linköping, Sweden) and designed for primary use in industrial applications. The polymer powder is fully derived from bio-based sources and exhibits a density of 0.65 g/cm^3^, as reported by the supplier’s technical datasheet. The gray appearance is inherent to the polymer’s natural coloration and is not due to the addition of pigments.

The PA11 powder was processed via SLS to selectively fuse the powder in nitrogen atmosphere using a Wematter Gravity SLS 300 (Wematter, Linköping, Sweden) equipped with a 40 W CO_2_ laser and a printing platform with a volume of 300 × 300 × 300 mm^3^ for printing large components for industrial use. The optimization of the printing parameters was carried out through a comprehensive preliminary set of tests, which involved an initial analysis of the PA11 printability based on the parameters reported in similar works already published [10], followed by methodical optimization of the main settings for the manufacturing of the desired parts.

The optimization process focused on identifying the parameter combination that ensured good powder spreading, uniform layer fusion, and the production of defect-free specimens with high dimensional accuracy and surface quality. The printing parameters were tuned after each single print by means of a trial-and-error approach to minimize porosity formation and avoid part distortion until the best resolution was achieved. The processing temperature was carefully monitored by checking the build chamber temperature via a thermocouple positioned inside the chamber to ensure uniform thermal conditions during the entire SLS process. The powder bed temperature was set at around 187 °C, based on the sintering window identified through DSC characterization in the range between 170 °C and 190 °C.

Furthermore, the laser power was set at 27 W and the scanning speed at 12 mm/h. The layer thickness was fixed at 0.1 mm with the infill density at 100% for all the PA11 parts to ensure highly dense structures. One hundred warming layers were also used for each print, representing the number of consecutive layers without any laser hatching.

These preliminary layers help to minimize temperature gradients within the build chamber and reduce thermal distortion, thereby improving layer adhesion and dimensional accuracy. The key processing parameters are listed in Table 1.

### 2.2. Characterization Techniques

The microstructures of the neat PA11 powder and the 3D-printed parts were investigated using a Phenom™ XL Scanning Electron Microscope (Thermo Fisher Scientific Inc., Waltham, MA, USA) at a voltage of 15 kV after metallization with platinum of the neat powder and the polymeric fracture surface of the 3D-printed specimen. The instrument was equipped with a SEM-energy dispersive spectroscope (EDS).

The particle size and circularity of the PA11 powder was evaluated by analyzing thousands of particles using an automated analyzer, Morphology 4 (Malvern Panalytical, Malvern, Worcestershire, UK), to obtain a statistical assessment of the particle size and shape, followed by using image processing software to capture high-resolution images of each particle and realize the powder landscape.

Thermogravimetric analysis (TGA) was performed on the neat PA11 powder and on the 3D-printed samples, using a temperature range of 25° to 800 °C, a heating rate of 10 °C/min, and a gas flow of 50 mL/min. Measurements were performed under both air and argon atmospheres, employing a Mettler Toledo TGA 851e Instrument (Mettler Toledo International Inc., Columbus, OH, USA).

Differential scanning calorimetry (DSC) analyses were conducted on the PA11 powder and the 3D-printed samples using a Hitachi NEXTA DSC 600 (Hitachi, Saitama, Japan) from −30 to 250 °C with a heating/cooling rate of 10 °C/min under nitrogen flow of 50 mL/min. Two heating cycles and one cooling cycle were performed.

The crystallization degree (*X_c_*) of the neat PA11 powder and the printed part was calculated by using the well-known Equation (1) [10,18]:

(1) $$X_{c}=\frac{{∆H}_{m}}{{∆H}_{m}^{0}}\times 100$$

where ${∆H}_{m}$ and ${∆H}_{m}^{0}$ are the enthalpy of melting experimentally evaluated and the melting enthalpy of the fully crystalline PA11 (226.4 J/g), respectively [15,19].

The true density (*ρ*) of the PA11 powder was calculated at room temperature using an Ultrapyc 5000 gas pycnometer (Anton Paar GmbH, Graz, Austria) with helium as the probe gas, following the ASTM B923-20 standard [20], to study the flowability of the powder. Five consecutive measurements, within a tolerance of 0.005%, were performed to guarantee the accuracy of the experimental results.

The flowability of the PA11 powder particles was evaluated by determining the packing factor (*φ*) and the Hausner ratio (*HR*), using a 25 mL graduated cylinder, according to a simplified method based on the ASTM D7481 standard [21] already reported in the literature [22]. *φ* and *HR* were calculated using Equations (2) and (3):

(2) $$\varphi =\frac{\rho_{bulk}}{\rho }$$

(3) $$HR=\frac{\rho_{tap}}{\rho_{bulk}}$$

where *ρ_bulk_* is the apparent density, *ρ* is the true density, and *ρ_tap_* is the tapped density of the powder, respectively. Before each test, the PA11 powder was oven-dried to eliminate the influence of the water that eventually absorbed onto the particles’ surface.

X-ray diffraction analyses were performed using an XRD Panalytical Empyrean Diffractometer (Malvern Panalytical, Malverm Worcestershire, UK) with a Bragg–Brentano configuration and Cu-K radiation (λ = 0.15406 nm), enabling the measurement of the d-spacing of multiple planes of different orientations. The data were collected over a 2θ range of 5–50°, with the instrument operating at 40 kV and 40 mA.

The bulk density of the 3D-printed samples was also evaluated through the non-destructive Archimedes method according to an ASTM B962–17 standard [23]. Since the tested material has a density close to 1 g/cm^3^, isopropyl alcohol (*ρ* = 0.785 g/cm^3^) was used as the liquid instead of distilled water. Five different specimens were tested to obtain statistically significant results.

The porosity was then calculated following Equation (4):

(4) $$P=\frac{\rho -\rho_{sls}}{\rho }\times 100$$

where *ρ_sls_* is the density of each 3D-printed sample and *ρ* is the true density of the starting PA11 bio-based powder previously measured using the gas pycnometer [10].

The *ρ_sls_* density values of the 3D-printed PA11 specimens were calculated using Equation (5) considering the total porosity present in the polymeric material (Total porosity density):

(5) $$\rho_{sls}=\frac{W_{air}\cdot \rho_{liq}}{W_{fin}-W_{liq}}$$

and Equation (6) considering the porosity closed (Closed porosity density):

(6) $$\rho_{sls}=\frac{W_{air}\cdot \rho_{liq}}{W_{air}-W_{liq}}$$

where *W_air_* is the mass of the samples in air, *W_liq_* is the mass of the sample immersed in the liquid, *W_fin_* is the mass of the sample after being extracted from the liquid, and *ρ_liq_* is the density of the isopropyl alcohol in which the samples are immersed.

Dynamic mechanical analysis (DMA) was carried out from 0 to 150 °C by using an Anton Paar MCR 702e Multi Drive Rheometer (Anton Paar GmbH, Graz, Austria), equipped with a chiller GCU 20. The tests were conducted by applying uniaxial sinusoidal stress with an amplitude of 1 N and a frequency of 1 Hz on rectangular specimens (50 × 10 × 2 mm) with a thickness of 2 mm.

The mechanical properties of the 3D-printed samples, such as the elastic modulus, ultimate tensile strength, and elongation at break percentage, were investigated by tensile testing according to an ISO 527-2 standard [24]. Tests were performed using an Instron 5966 tensile testing machine (Instron 5966, Instron Corp., Norwood, MA, USA), equipped with a 2 kN load cell and pneumatic grips, applying a deformation rate of 1 mm/min with a grip separation of 50 mm. The tested specimens were dog-bone-shaped (type 5A) with a gauge length of 26 mm, width of 4 mm, and thickness of 2 mm.

To assess the effectiveness and precision of the SLS process, a nominal-to-actual geometry comparison was conducted on four components of a hose reel: the shaft holder, cover spring box, half-case, and shaft spring. This analysis involved comparing the original CAD models with the reconstructed 3D data obtained via micro-computed tomography (X-CT) using a Phoenix v|tome|x S240 scanner (GE Baker Hughes–Waygate Technologies, Wunstorf, Germany). The scanning parameters were set to 210 kV voltage, 110 µA, and 100 ms exposure timing, and 1500 for the number of projections.

All X-CT data were reconstructed into 3D models using datos|reconstruction software (version 2.8.0—RTM), and the subsequent visualization and analysis were carried out with VG Studio Max (version 3.4, Volume Graphics, Hexagon Metrology–Volume Graphics, Heidelberg, Germany). Surface determination was conducted using the Advanced (classic) method, which relied on histogram-based contour identification with Automatic material definition at a 50% iso-value threshold. Based on the defined surfaces, the nominal/actual comparison module within VG Studio was used to evaluate the dimensional accuracy of the printed parts.

## 3. Results and Discussion

### 3.1. Characterization of the PA11 Powder

**Figure 1:**
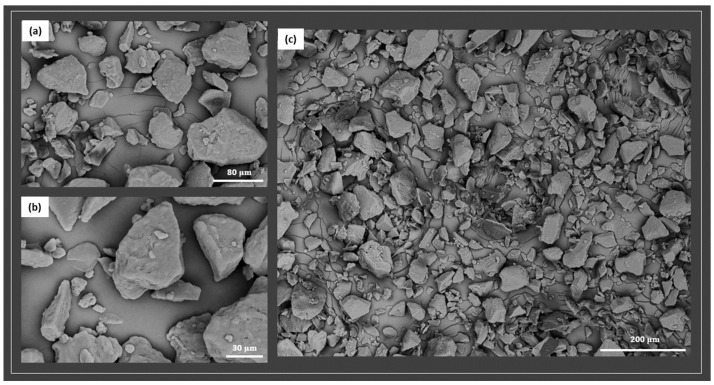
SEM images of PA11 powder at different magnifications, 2000× (**a**), 1000× (**b**), and 300× (**c**), respectively.

The morphology of the pristine PA11 powder was first investigated by means of SEM/EDS analysis. Figure 1 shows the shape and dimension of PA11 particles at different magnifications: 2000× (a), 1000× (b), and 300× (c).

The micrographs show the presence of particles with irregular shapes of sizes lower than 100 microns, indicating that the powder has characteristics in line with the required powder size range for ideal SLS processing [10,18].

Traces of small silica nanoparticles, less than 4 wt.%, were also detected within the PA11 powder by EDS analysis, presumably added by the supplier to increase the flowability of the PA11 in the SLS 3D printer.

Moreover, granulometric analysis was performed on the powder to obtain a representation of its morphological features in terms of particle size distribution and diameter. Table 2 lists the main PA11 particle parameters, such as diameter, aspect ratio, and circularity.

The results provide evidence that PA11 can be processed via SLS, showing the ideal particle dimension with an average diameter lower than 80 microns, and a circularity close to 1. The circularity refers to the degree to which the individual powder particles approximate a perfect spherical shape. The circularity close to 0.9 clearly indicates the PA11 particles are nearly spherical. This is a morphological parameter commonly used to assess the quality of powders used in SLS. This parameter also implies good flowability for guaranteeing the spreading of the powder layer by layer into the 3D printer and leading to the realization of the desired parts [22].

For this reason, the PA11 powder density and flowability were evaluated. The true density of the PA11 powder was measured using a gas pycnometer and found to equal 1.063 ± 0.002 g/cm^3^ (see Table 3), a value slightly higher with respect to the value declared in the supplier datasheet for the PA11 powder, 0.65 g/cm^3^. This difference can be due to the different types of measurement employed for the density evaluation [25]. The value measured using a gas pycnometer refers to the true density of the polymeric material, which measures the powder’s density without accounting for voids.

The flowability and packing factor of the PA11 powder were also calculated, being two important key parameters for a SLS-based process. In fact, a powder with high circularity and smooth surfaces typically has good flowability and thus achieves higher packing density for obtaining high-quality, dimensionally accurate, and mechanically reliable SLS parts, especially when working with polymer powders such as PA11.

The Hausner ratio was found to be 1.17 ± 0.01, indicating good powder flowability, with a packing factor of 0.46 ± 0.01. This is within the acceptable range for polymer powders used in SLS, like polyamides, which generally show packing factors between 0.40 and 0.55, depending on the particle shape, size distribution, and surface roughness.

Considering these data, the PA11 powder spreading and layer deposition were expected to be stable in the SLS process, as already discussed in literature that underlines that PA11 powders, when adequately processed and possessing an appropriate particle size distribution, exhibit good flowability and high packing density. The packing and flowability values of PA11 powder align well with previously reported values for a commercial PA12 powder [22]. Although these kinds of characterizations indirectly reflect the powder’s flowability and spreadability, it is possible to conclude that the properties of PA11 powder ensure uniform layer formation during the SLS process.

**Figure 2:**
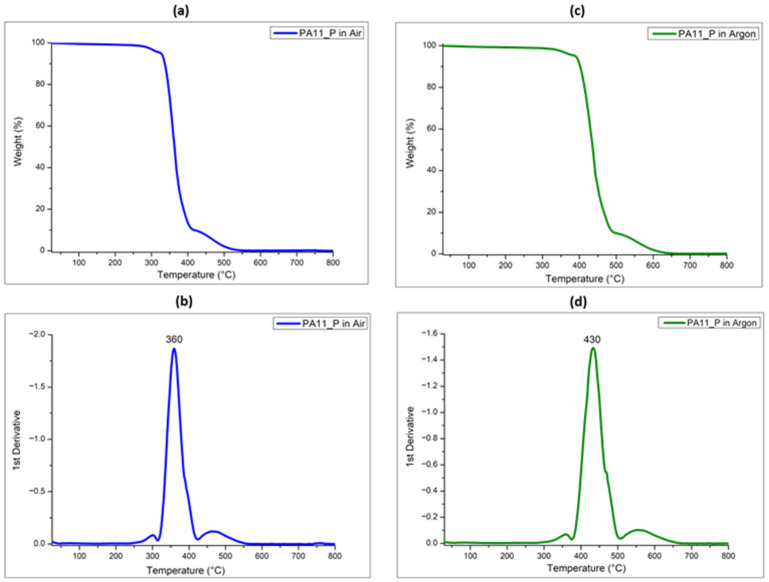
TG and derivative TG (DTG) curves of PA11 powder (PA11_P) performed in air (**a**,**b**) and in argon (**c**,**d**).

Finally, the thermal behavior of the PA11 powder was assessed by using TGA measurements performed from room temperature up to 800 °C, both in air (Figure 2a) and argon (Figure 2c). The thermal decomposition of the neat PA11 powder involves one single step, where the temperature at which the maximum degradation peak occurs in air, evaluated by the derivative mass loss data, was 360 °C, with zero residue at 800 °C, as reported from Figure 2b. The decomposition behavior of the polymer powder, evaluated in inert atmosphere using an argon flow, also reveals one degradation peak occurring at higher temperature, 430 °C, as visible in Figure 2d, leading to an ash residue of 0.7 wt.% evaluated at 800 °C.

This difference can be explained considering that the presence of air leads to a more aggressive oxidative degradation process, as oxygen promotes the breaking of the amide bonds, causing the polymer to decompose into volatile products, such as amines and acids [15]. The degradation temperature shifts toward higher temperatures in the presence of inert atmosphere because the PA11 polymer’s molecular structure tends to break down more uniformly in argon by means of chain scission reactions, producing fewer by-products in a more controlled process [15].

**Figure 3:**
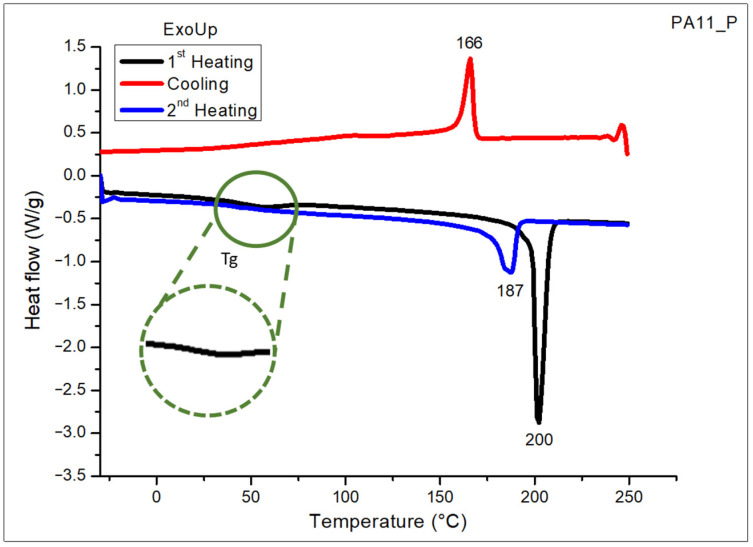
DSC curves of PA11 powder (PA11_P) performed in nitrogen.

The thermal transitions and the degree of crystallinity of the PA11 powder were evaluated via DSC performed in nitrogen atmosphere. The DSC curves of PA11 powder relative to the cooling and the second heating scans, after erasing the thermal history of the polymer with the first heating run, are illustrated in Figure 3.

It can be observed that PA11 presents a single crystallization peak centered at 166 °C, and a melting temperature with a maximum at 187 °C followed by a small shoulder, from a rearrangement of the polymeric structure after crystallization due to the different crystalline structures of PA11 depending on temperature and cooling conditions [19]. The shoulder peak corresponds to the α′ crystalline form of PA11 while the second peak is assigned to the γ crystalline form for the neat PA11 [19,22,25,26].

Moreover, from the DSC analysis, it was also possible to determine the glass transition temperature (T_g_) of PA11, evaluated as the midpoint of the change in the slope of the baseline curve, which was found to be close to 58 °C. This is more clearly visible in the green enlarged section of Figure 3 relative to the first heating scan.

The crystallinity of the PA11 matrix was also measured as the ratio between the enthalpy of polymer crystallization and the theoretical value of the fully crystalline polymer matrix. The fusion enthalpy for PA11 evaluated from the analysis of the area under the melting curve was 98.4 J/g, allowing us to estimate the crystallization degree of the semicrystalline polymeric powder, considering 100% crystalline PA11 to have an enthalpy value of 226.4 J/g based on the value reported in the available literature [15,19]. Thus, the value of the degree of crystallinity percentage calculated for the PA11 powder employed in the present research was 43%, as reported in Table 4.

The polymer phase transitions play a key role in also defining the sintering window of the PA11 powder for SLS applications, which is determined as the temperature range between the crystallization onset and the melting point onset temperatures, as reported in the literature [27,28]. The sintering window for the PA11 was found to be in the range between 166 °C and 190 °C, underlining that the PA11 powder can be successfully processed by SLS technology in that temperature range. The crystalline structure of PA11 was also studied using an X-ray diffractometer.

**Figure 4:**
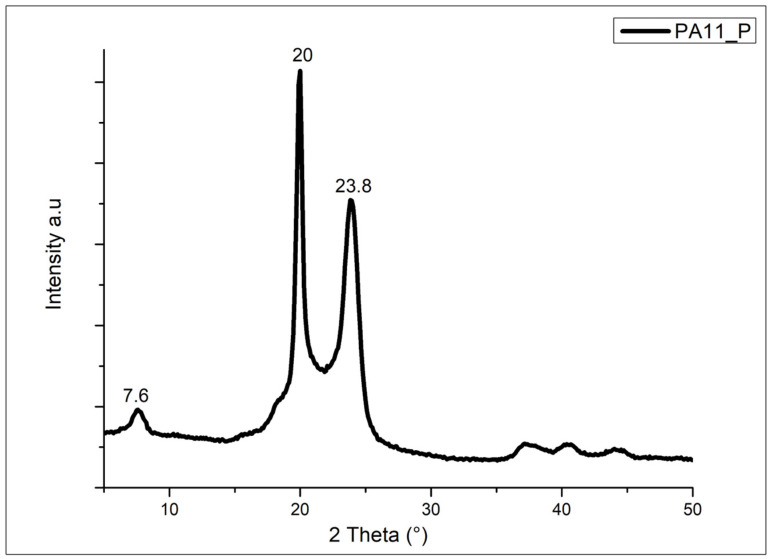
XRD spectrum of the neat PA11 powder (PA11_P).

As already discussed, PA11 presents two different crystalline structures during the second melting (γ and α′ forms). It can be noted that these structures were obtained after a controlled crystallization process followed by a melting process. Figure 4 shows the XRD spectrum of the neat PA11 powder.

The X-ray diffraction profile of the PA11 powder reveals the presence of three characteristic peaks at 7.6°, 20°, and 23.8°, respectively, attributed to the (001), (100), and (010/110) planes, indicating the presence of the triclinic α-form of PA11 [19,22,25,26,27,28].

### 3.2. Density Tests of PA11 Printed Parts

The density values of the PA11 printed parts were experimentally evaluated by using the buoyancy method based on Archimedes’ principle, which allows us to evaluate the difference in buoyancy of the specimen measured in air and submerged into isopropyl alcohol, used as a reference liquid with known density. Archimedes’ method was used to estimate a global density value relative to the reference liquid, and the density was compared to the material’s nominal reference density [10,25].

Table 5 reports the average values of density and porosity of the 3D-printed PA11 parts estimated by using Equations (4)–(6).

The density values of the closed and total porosity indicate a total porosity of 0.8%, highlighting the excellent densification and definition of the PA11 samples obtained via SLS. This low value of porosity can be considered a positive characteristic of the 3D-printed specimens realized, underlining that the finished parts are solid and dense with very few voids, which can result in improved mechanical properties and stability for industrial applications.

### 3.3. Microstructure of PA11 Printed Parts

**Figure 5:**
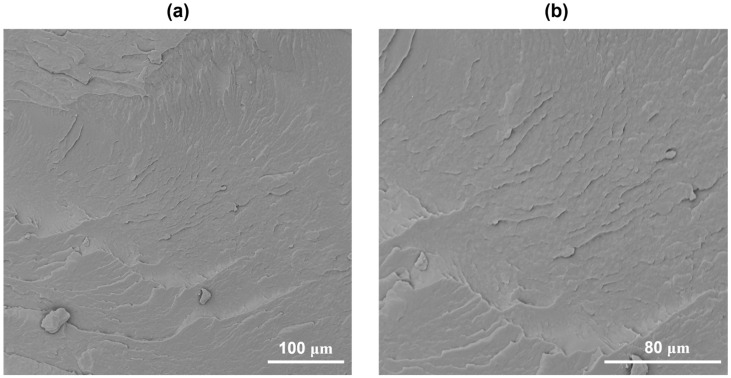
SEM micrographs of the cryo-fractured surface of 3D-printed PA11 specimens at 500× (**a**) and 1000× (**b**).

The microstructure at different magnifications of the 3D-printed parts manufactured via SLS was investigated by using SEM analysis of the parts’ fracture surfaces, as shown in Figure 5.

The SEM images clearly show that PA11 powder was melted into well-densified parts with a very smooth surface and no evidence of voids, confirming the results of porosity, as previously discussed. The 3D-printed components show minimal porosity, good powder sintering, and strong internal bonding of the material layers. This represents a crucial aspect for ensuring the structural integrity, thermal stability, and mechanical performance of the parts for future industrial applications.

### 3.4. Thermal Properties of PA11 Printed Parts

**Figure 6:**
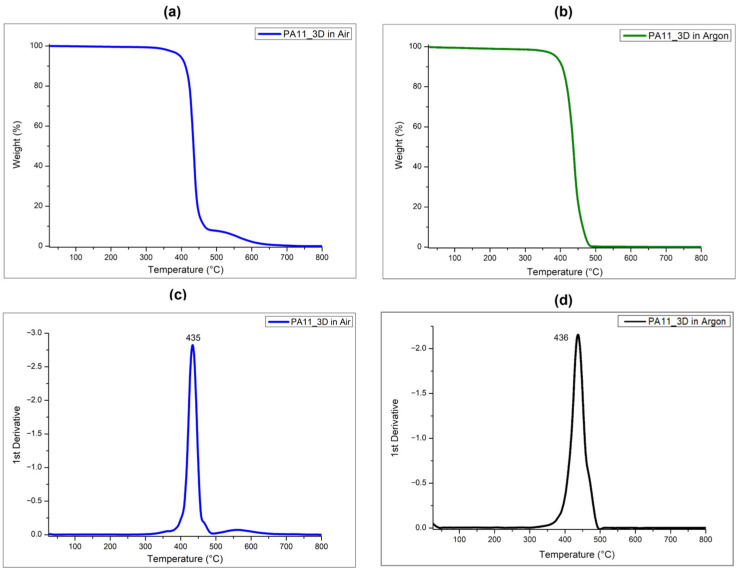
TG and derivative TG (DTG) curves of PA11 printed specimens (PA11_3D) performed in air (**a**,**b**) and in argon (**c**,**d**).

Thermal properties of the 3D-printed PA11-based specimens were evaluated by using TGA and DSC. Figure 6 reports the thermograms in air (Figure 6a) and argon (Figure 6b) and the relative first derivative curves of the PA11 samples obtained via SLS.

The behaviors of the curves are almost the same for both the two series of measurements performed in oxidant and inert conditions, and evidence one degradation peak with a maximum at around 435 °C. This result demonstrates that the sintering process within the SLS 3D printer does not have effects on the thermal stability of the polymer that remains the same as the untreated powder.

**Figure 7:**
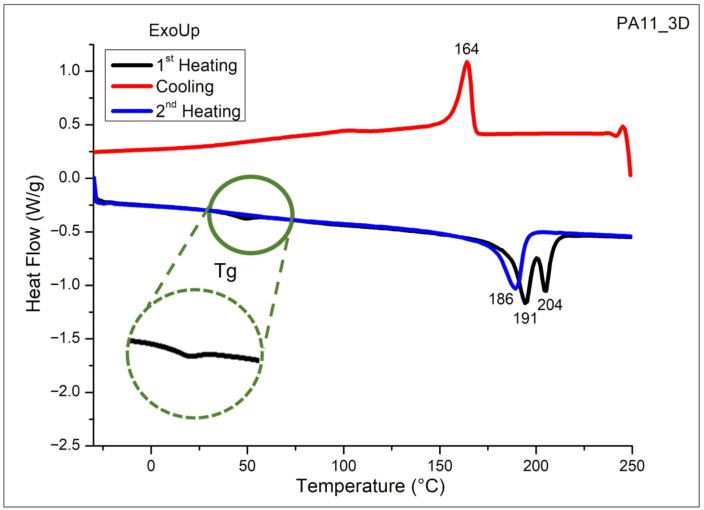
DSC curves of PA11 3D-printed specimens performed in nitrogen.

While the sintering process may not drastically affect the thermal stability of the polymer, it can influence its thermal properties. Therefore, DSC analysis was also performed on the PA11 printed samples obtained via SLS, as shown in Figure 7.

The DSC curves of the 3D-printed specimens show, as for the powder, a crystallization peak at 164 °C and a melting peak at 186 °C, while the glass transition temperature was found to be around 50 °C, as more evident from the green enlarged section of Figure 7. The degree of crystallinity evaluated using Equation (1) was 35%, a value slightly lower with respect with the neat PA11 powder crystallinity (43%).

This result evidenced that during the printing process, the polymeric material was heated to a molten state and then rapidly cooled to solidify layer by layer according to the desired CAD model. However, the cooling rate is much faster compared to the traditional processing methods and does not provide enough time for the PA11 polymer chains to align in an orderly crystalline structure, leading the formation of more amorphous regions, resulting in a reduction in the degree of crystallinity [26,29].

**Figure 8:**
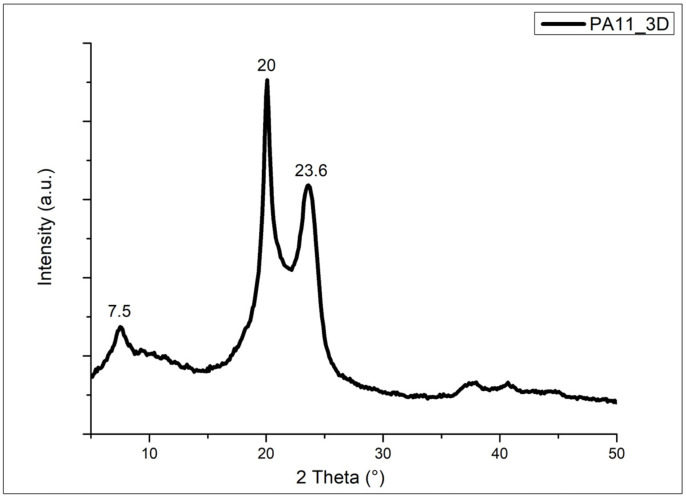
XRD spectrum of a PA11 3D-printed sample (PA11_3D) obtained by SLS.

The crystalline structure of the PA11 was also confirmed by XRD analysis on a bulk sample obtained by SLS, as visible in Figure 8.

The diffraction pattern of the 3D-printed specimen has a profile like that of the neat PA11 powder, showing the three main peaks at 7.5°, 20°, and 23.6°, indicating the presence of the α crystalline phase structure, as previously seen for the PA11 powder. This result also underlines that the sintering process does not significantly influence the crystalline structure of the polymer matrix. The printed part remains relatively similar in crystallinity to the initial powder.

### 3.5. Dynamic-Mechanical Properties of PA11 Printed Parts

**Figure 9:**
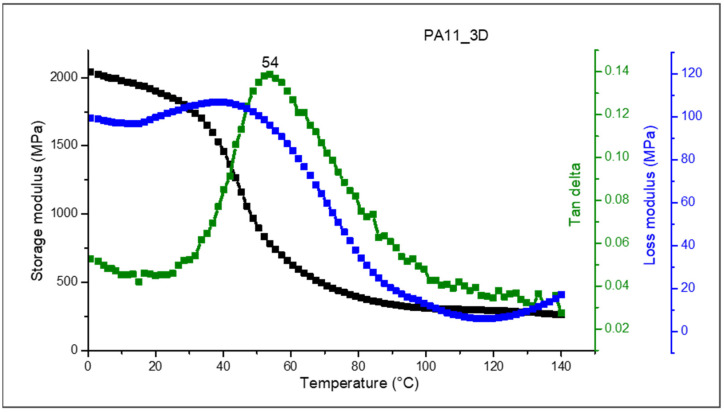
DMA curves of PA11 3D-printed specimens (PA11_3D) performed in tensile mode: storage modulus (black curve), loss modulus (blue curve), and tan delta (green curve).

DMA was performed to obtain indications about the 3D-printed PA11′s viscoelastic properties as a function of temperature. The trends of the extensional storage modulus (E′), the loss modulus (E″), and the tan δ values as a function of temperature are reported in Figure 9.

The storage modulus represents the elastic behavior of the polymeric material’s deformation, indicating its stiffness, while the loss modulus indicates the viscous component of the deformation, reflecting the energy dissipated as heat. As visible from Figure 9 (black curve), at lower temperatures, PA11 exhibits a high storage modulus, as the material behaves more like a glassy polymer. The storage modulus in the glassy region at 0 °C is 2040 MPa. As the temperature increases, PA11 softens, and the storage modulus strongly decreases, especially near the T_g_. Once the temperature is over the T_g_, the storage modulus values are relatively low, indicating a high mobility of the polymer molecules corresponding to the amorphous phase of the PA11 [30].

From DMA, it is also possible to determine the PA11 glass transition temperature by evaluating the maximum of the tan delta curve. The T_g_ value of the PA11 printed sample was found at 54 °C (green curve), providing insight into the temperature at which PA11 transitions from a glassy to a rubbery state. A different behavior was observed for the loss modulus of PA11 (blue curve), which is 108 MPa in the glassy region at 0 °C, and increases near the glass transition temperature, where the polymeric chains’ mobility increases [30]. The DMA results underline that the PA11-based printed specimens have properties suitable for many industrial applications including functional parts.

### 3.6. Tensile Properties of PA11 Printed Parts

**Figure 10:**
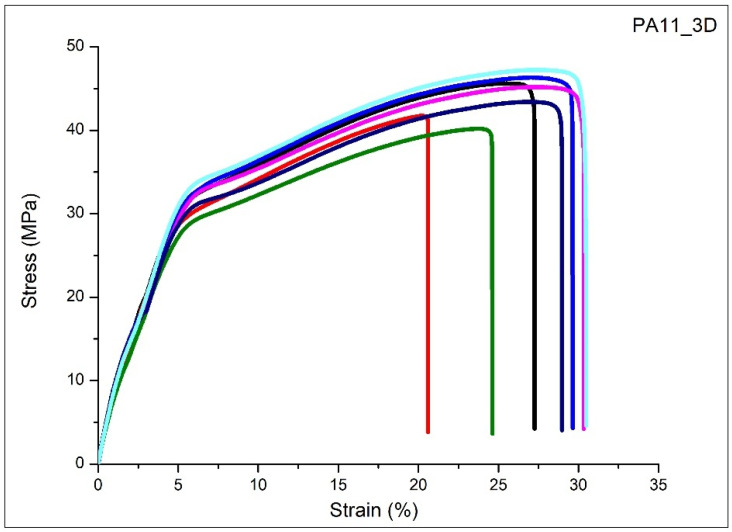
Tensile stress–strain curves of seven PA11 3D-printed specimens (PA11_3D).

Finally, the mechanical properties of the PA11 printed parts were evaluated by using tensile tests. The results concerning the elastic modulus, tensile strength, and deformation were obtained by analyzing the tensile stress–strain curves at room temperature, as shown in Figure 10.

The PA11 dog-bone specimens show a quasi-brittle behavior where, after the primary elastic deformation, the samples undergo yielding followed by a plastic deformation region. The average values of the elastic modulus, ultimate tensile strength, yield strength, and elongation at break percentage are summarized in Table 6 with their standard deviations.

The Young’s modulus measures the polymer stiffness; the value reveals that the PA11-based 3D samples are moderately stiff and can resist deformation while still remaining flexible. Interestingly, the yield strength (44 MPa) is almost equal to the ultimate tensile strength (45 MPa). In the present study, the inflection occurs at approximately 30–33 MPa, whereas the 0.2% offset method provides a yield strength of about 44 MPa, fully consistent with the expected mechanical response of engineering polymers like PA11.

In materials such as PAs, which typically do not exhibit a well-defined yield plateau, the offset procedure often yields a value that lies very close to the ultimate tensile strength. In this case, the ultimate tensile strength is 45 MPa, only 1 MPa higher than the yield strength, which is coherent with the characteristic stress–strain behavior of semicrystalline polymers [31]. PA11 does not have a large plastic deformation plateau, meaning it starts to fail soon after yielding, evidencing a typical behavior of SLS polymers where interlayer bonding limits ductility, and also revealing the deformation percentage (27%).

These mechanical properties indicate PA11 is suitable for lightweight, moderately loaded parts where flexibility and energy absorption are important, but not for applications requiring very high strength or rigidity. In general, PA11-based samples printed via SLS show values of the elastic modulus, tensile and yield strengths, and elongation at break lower than injection-molded parts, referring to the technical datasheet and the available literature [32].

This can be explained considering that SLS parts can show a higher potential porosity, which can affect their stiffness and impact the material’s overall rigidity. Moreover, the sintering process can also lead to anisotropic material properties, where the mechanical properties can vary depending on the part orientation [32].

However, this finding underlines that SLS can be considered as a useful method for PA11 processing, giving rise to the realization of functional parts for prototypes for industrial uses through an AM approach, guaranteeing good mechanical performance and reducing the environmental footprint of the fabrication process.

### 3.7. Fabrication via SLS of Industrial Components for Prototype

A series of printing tests were first performed to assess the optimal printing parameters and the PA11 powder printability via SLS, by means of a trial-and-error approach. Consequently, several specimens were 3D-printed starting from simple rectangular and dog-bone shapes for the characterization tests up to more complex architectures to realize the hose reel prototype components for industrial applications.

**Figure 11:**
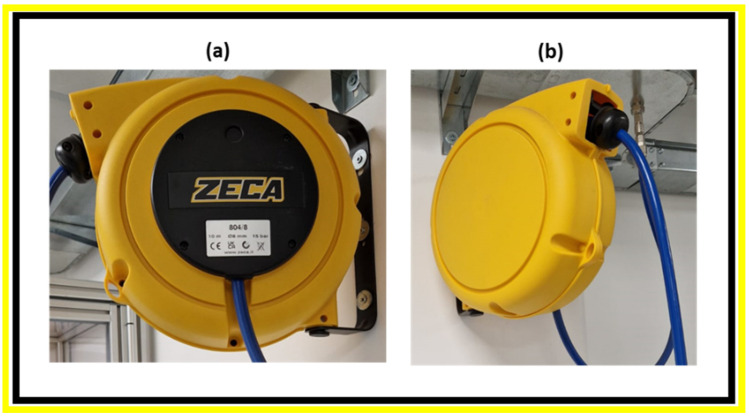
Commercial ZECA hose reel from different views: frontal (**a**) and side (**b**).

Figure 11 shows pictures from different points of view of a commercially available hose reel produced by the ZECA company and industrially obtained by injection molding.

**Figure 12:**
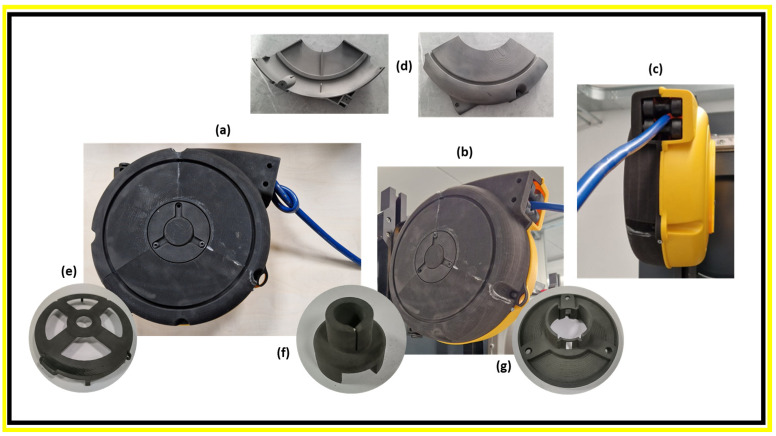
Different views of the hose reel prototype (**a**–**c**), assembled by using the half-cases (**d**), shaft spring (**e**), cover spring box (**f**), and shaft holder (**g**) realized via SLS using PA11.

The pictures in Figure 12 show the top and side views of the hose reel assembly (Figure 12a–c) obtained by SLS using PA11 powder. It also shows different components 3D-printed via SLS to fabricate the final prototype, such as half-cases (Figure 12d), a shaft spring (Figure 12e), a cover spring box (Figure 12f), and a shaft holder (Figure 12g). The half-cases were obtained by printing three different parts and then assembling them, as shown in Figure 12b.

Well-consolidated parts with a very high level of definition were successfully fabricated considering their large and complex structure. The 3D-printed components also display a smooth surface finish, contributing to both aesthetic quality and functional performance. These are all important characteristics that are highly valued in industries such as aerospace, automotive, and high-performance engineering, especially for components exposed to high stress, like for hose reel applications. The ability to achieve such high levels of definition combined with material consolidation provide the opportunity to produce better-performing and higher-quality parts for functional industrial use.

**Figure 13:**
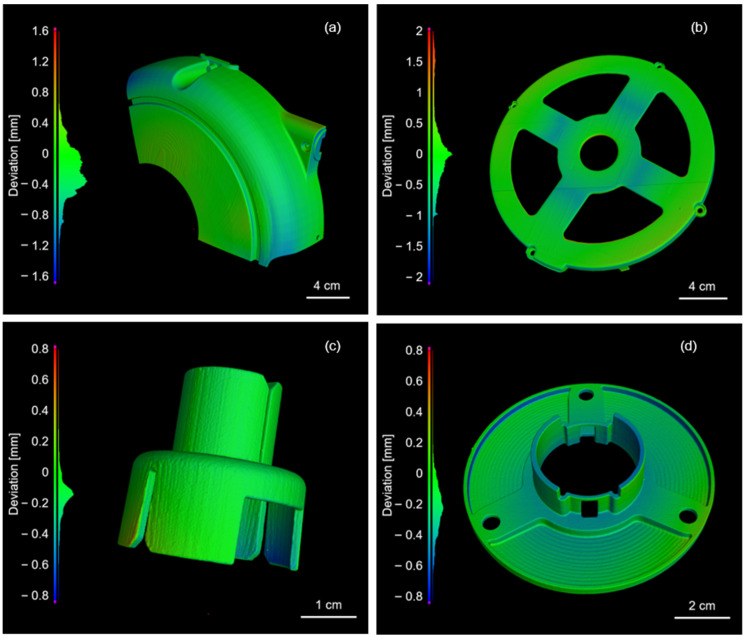
Nominal-actual dimensional deviation analysis for different components: half-case (**a**), shaft spring (**b**), cover spring box (**c**), and shaft holder (**d**).

To better evaluate the effectiveness of the SLS manufacturing process used for the hose reel components, a comparison between nominal and actual dimensions was carried out on four components produced by SLS: the half-cases (Figure 13a), shaft spring (Figure 13b), cover spring box (Figure 13c), and shaft holder (Figure 13d).

Figure 13 presents chromatic deviation maps illustrating the dimensional accuracy of the printed components relative to their original CAD models. Dimensional accuracy is not only of primary scientific concern but is also crucial for practical functionality. Since all four components must be assembled for their intended use, ensuring dimensional fidelity is essential.

To quantitatively assess accuracy, the absolute cumulative deviation at the 95th percentile was used. This metric indicates the extent of the component’s surface that falls within a specific deviation threshold. The half-case exhibited a 95% cumulative deviation of 0.72 mm, while the shaft spring showed a higher deviation of 1.5 mm. In contrast, the cover spring box and the shaft holder displayed better dimensional accuracy, with cumulative deviations of 0.37 mm and 0.48 mm, respectively.

It is noteworthy that the components exhibiting the largest dimensional deviations are also those with the greatest overall size, as reported in the previous literature [33].

These deviations could potentially be reduced by appropriately rescaling the original design dimensions. In most cases, deviations were concentrated around connecting features or regions with varying inclinations, in good agreement with previous studies [34,35]. An exception is the shaft spring (Figure 12e), where the highest deviation was observed near the central hole. This discrepancy is not attributed to the SLS printing process, but rather to the functional testing performed on the component prior to X-CT scanning, which can cause localized downward deformation in a structurally weaker area.

Nevertheless, all components were successfully assembled and functionally tested, confirming that the dimensional accuracy achieved with commercial PA11 powder and the Wematter Gravity SLS 300 system was sufficient for industrial application. These results demonstrate that the 3D-printed hose reel parts reliably reproduced the intended complex geometries with the necessary resolution and fit for their final use.

## 4. Conclusions

Selective laser sintering was used as an AM technology to develop a functional prototype for industrial applications, like hose reels, by using a bio-based PA11 powder.

Firstly, the powder flowability and printability were studied by identifying the best printing parameters to optimize the SLS printing process. Wide characterization of the polymeric powder was carried out to investigate its thermal behavior and thermal stability, sintering window, morphology, and porosity, all key factors for the 3D printing process.

The combination of fine particle size (45 microns), near-spherical morphology (circularity close to 1), and favorable flowability (Hausner ratio of 1.17) of the PA11 contribute to homogeneous powder spreading and efficient layer consolidation during the SLS process.

Consequently, such characteristics are essential in achieving high part quality, dimensional accuracy, and overall process stability.

Several larger printed components with very complex structures, custom features, and functional capabilities were successfully fabricated for the first time using PA11 for SLS applications according to the authors’ best knowledge.

The characterization results of the 3D-printed specimens evidenced that the sintering process occurring within the SLS 3D printer does not significantly affect the thermal degradation and thermal stability of the PA11 powder, as shown by DSC and TGA measurements. XRD and DSC analyses also led us to study the crystallinity of the PA11 before and after the printing process. These findings were confirmed by the morphological evidence coming from SEM analysis and the porosity tests, which underline that the PA11 powder particles are well-sintered and show very low porosity. The PA11 3D-printed specimens were also characterized from viscoelastic and mechanical points of view. The DMA and tensile testing results clearly reveal that PA11 powder processed by SLS led to fabricated printed components with high printing resolution and dimensional accuracy and with mechanical properties suitable for the industrial application of hose reel prototyping.

The SLS-fabricated PA11 samples exhibited an average elastic modulus of 925 MPa, similar ultimate tensile strength and yield strength values, 44 and 45 MPa, and an elongation at break of 27% confirming the material’s ductile behavior. Based on this mechanical performance, PA11 can be considered appropriate in applications involving lightweight structures and moderate loads, particularly where flexibility and impact energy absorption are beneficial. However, its use is limited in contexts that require high mechanical strength or rigidity. The parts obtained reveal that SLS enables the production of lightweight components with complicated geometry, reducing material usage while maintaining part integrity and strength.

Moreover, the PA11 results are ideal for functional prototypes being both strong and flexible, allowing the replication of the material properties of end-use parts to remain customizable and cost-effective. The customization achieved through the AM approach was also valorized and enhanced using bio-based PA11 powder, decreasing raw material consumption and reducing the environmental impact.

## Figures and Tables

**Table 1:** Selective laser sintering main printing parameters for PA11.

Process Parameters	
Laser power [W]	27
Scan speed [mm/s]	0.0033
Layer height [mm]	0.1
Powder bed temperature [°C]	187
Sintering window [°C]	170–190
Number of warming layers	100

**Table 2:** PA11 particle size distribution, diameter, and circularity evaluated by granulometric analysis.

Sample	Diameter (µm)	Aspect Ratio	Circularity
PA11	D[*n*, 0.1]:9.5	D[*n*, 0.1]:0.5	D[*n*, 0.1]:0.7
	D[*n*, 0.5]:20.0	D[*n*, 0.5]:0.7	D[*n*, 0.5]:0.9
	D[*n*, 0.9]:45.2	D[*n*, 0.9]:0.9	D[*n*, 0.9]:0.9

**Table 3:** Density and flowability values of the PA11 powder.

Sample	True Density (g/cm^3^)	Apparent Density (g/cm^3^)	Tapped Density (g/cm^3^)	Hausner Ratio (HR)	Packing Factor
PA11_P	1.063 ± 0.002	0.50 ± 0.01	0.58 ± 0.01	1.17 ± 0.01	0.46 ± 0.01

**Table 4:** Thermal properties of PA11 powder (PA11_P) and printed samples (PA11_3D) evaluated by DSC.

Sample	T_g_^a^ (°C)	T_m1_ ^a^ (°C)	T_m2_ ^b^ (°C)	ΔH_m_ ^a^ (J/g)	T_c_ ^a^ (°C)	X_c_ ^c^ (%)
PA11_P	58	200	187	98.4	166	43
PA11_3D	50	191/204	186	78.7	164	35

^a^ Transition temperature obtained by DSC performed in nitrogen analyzing the first heating and cooling scans; ^b^ Transition temperatures obtained by DSC performed in nitrogen analyzing the second heating scan; ^c^ Degree of crystallinity calculated using Equation (1).

**Table 5:** Density and porosity values of the 3D-printed PA11 specimens (PA11_3D).

Sample	Density (Closed Porosity) (g/cm^3^)	Density (Total Porosity) (g/cm^3^)	Porosity (%)
PA11_3D	1.050 ± 0.004	1.054 ± 0.004	0.8

**Table 6:** Tensile properties of the 3D-printed PA11 specimens (PA11_3D).

Sample	Elastic Modulus (MPa)	Ultimate Tensile Strength (MPa)	Yield Strength (MPa)	Elongation at Break (%)
PA11_3D	925 ± 52	45 ± 3	44 ± 2	27 ± 4

## Data Availability

The original contributions presented in this study are included in the article. Further inquiries can be directed to the corresponding author.
